# Identification of an Integrase That Responsible for Precise Integration and Excision of *Riemerella anatipestifer* Genomic Island

**DOI:** 10.3389/fmicb.2019.02099

**Published:** 2019-09-20

**Authors:** Ying Wang, Yang Zhang, Yijie Cui, Zhijian Sun, Zutao Zhou, Sishun Hu, Shaowen Li, Mei Liu, Xianrong Meng, Yuncai Xiao, Deshi Shi, Dingren Bi, Zili Li

**Affiliations:** ^1^State Key Laboratory of Agricultural Microbiology, College of Veterinary Medicine, Huazhong Agricultural University, Wuhan, China; ^2^Key Laboratory of Preventive Veterinary Medicine in Hubei Province, Wuhan, China; ^3^Key Laboratory of Development of Veterinary Diagnostic Products, Ministry of Agriculture of the People’s Republic of China, Wuhan, China

**Keywords:** *Riemerella anatipestifer*, genomic island, integrase, integration, excision, gene expression

## Abstract

*Riemerella anatipestifer* is a Gram-negative, pathogenic bacterium, which is harmful to poultry. However, the genomic islands (GIs) in *R. anatipestifer* are not well-studied. In this study, a 10K genomic island was predicted by the bioinformatics analysis of *R. anatipestifer* ATCC 11845, which is widely found in other *R. anatipestifer* genomes. We had first reported the genomic island integration and excision function in *R. anatipestifer*. We successfully constructed the integration plasmid by using the integrase and 53 bp attP elements. The 10K GI was found integrated at the 53 bp attB located in the Arg-tRNA of the *R. anatipestifer* RA-YM chromosome. We identified an integrase that helped in the precise integration and excision in *R. anatipestifer* and elucidated the molecular mechanism of the 10K genomic island integration and excision. Furthermore, we provided a new method for the gene expression and construction of complementary strain.

## Introduction

Genomic islands (GIs) are the segments of genomic DNA, which were originally known as pathogenicity islands (PAIs) in the 1980s. The GIs were first reported by Hacker while studying the virulence mechanisms in genetics and evolution of *Escherichia coli* (Hacker et al., 1997). They play a key role in prokaryotic genome plasticity. GIs are integrated into chromosomal loci, such as transfer RNA genes and protein-coding genes while retaining various cargo genes that potentially bestow novel functions on the host organism. The GIs also have integrase/recombinase genes that facilitate chromosome integration and excision (Hsiao et al., 2005). Recombinase belongs to two classical protein superfamilies, tyrosine recombinase family and serine recombinase family, which catalyze the formation of direct repeat sequences attL and attR at the flank of GIs to move the GIs. The excisionase will cut off the direct repeat sequence at both the ends of the GIs and form attB and attP (Williams, 2002).

There are two main requirements for the integration: functional element-encoded recombination enzymes and presence of short attachment sites, phage attachment site (attP) and bacterial attachment site (attB), recognized in site-specific recombination (Antonenka et al., 2005). Integration is catalyzed by an integrase protein (Int) mediating site-specific recombination between attP and attB, and generating direct repeats called attL and attR as products of the reaction (Singh et al., 2013). The integration of GIs into the bacterial chromosome and their excision can occur naturally (Ubeda et al., 2007). There are two modes of excision, excisionase-dependent, and excisionase-independent, but both require interaction with the integrase (Bergemann et al., 1995; Schubeler et al., 1997; Semsey et al., 1999; Mir-Sanchis et al., 2012).

The genomic islands carry multiple genes encoding a variety of biologically functional proteins related to virulence, antimicrobial resistance, and metabolic pathways (Ogier et al., 2010; Farrugia et al., 2015). In pathogenic bacteria, the virulence genes are carried by the GIs, and therefore, they are called PAIs. *Salmonella* pathogenicity islands (SPIs) and *Staphylococcal* pathogenicity islands (SaPIs) have been widely reported (Nieto et al., 2016; Novick and Ram, 2016). Some PAIs carry secretory systems, the type III secretory systems were coded by SPIs (Kim et al., 2018; Takaya et al., 2019) and type IV secretory systems were coded by *Helicobacter pylori* PAI (Horridge et al., 2017; Skoog et al., 2018). Moreover, GIs usually carry genes encoding hypothetical proteins whose functions are unknown. The GIs are the reservoirs of many new proteins, which can affect environmental adaptability (Dobrindt et al., 2004).

*Riemerella anatipestifer* is a Gram-negative, pathogenic bacterium, harmful to all kinds of poultry, such as ducks, turkeys, and geese (Segers et al., 1993; Leavitt and Ayroud, 1997). Till date, 33 genomic sequences of *R. anatipestifer* have been assembled, GIs have been reported (Hu et al., 2017; Zhu et al., 2019), but integration and excision GIs had not reported. In this study, we found a 10K genomic island in *R. anatipestifer* ATCC11845. Bioinformatics analysis revealed the presence of the 10K GI in 20 strains of *R. anatipestifer*. A tyrosine integrase was identified that could accurately perform both integration and excision in *R. anatipestifer*. This is the first report about an integrase-mediated integration and excision in *R. anatipestifer*, and the application of this integrase could mediate the expression of exogenous genes. This study laid the foundation for horizontal gene transfer and evolution of *R. anatipestifer* and provides a new method for gene expression in *R. anatipestifer*.

## Materials and Methods

### Genome-Wide Comparative Analysis of the 10K GI in *R. anatipestifer*

Thirty-three genomic sequences of *R. anatipestifer* have been obtained from NCBI^1^. IslandViewer 4 was used to predict GIs (Bertelli et al., 2017) and the artemis comparison tool (ACT) for comparing the 10K GIs (Carver et al., 2005). Homologous amino acid sequences were identified by searching the GenBank database using BLASTX (Marchler-Bauer et al., 2017). The nucleotide and protein sequence alignments were used for the construction of a phylogenetic tree using the neighbor-joining (NJ) method and were further analyzed using the MEGA v6.06 software (Wang et al., 2017).

### Plasmids, Bacterial Strains, and Growth Conditions

The plasmids and bacterial strains used in this study are listed in Table 1. *R. anatipestifer* ATCC11845 was purchased from the microbial preservation center (Guangzhou, Guangdong), whereas *R. anatipestifer* RA-YM was a lab collection (Zhou et al., 2011). *R. anatipestifer* was grown in trypticase soy broth (TSB) or on agar plates (Difco Laboratories, United States) at 37°C in 5% CO_2_. *E. coli* X7213 was cultured in Luria Bertani (LB) broth containing 100 μg/mL diaminopimelic acid (DAP), with shaking at 37°C overnight (Roland et al., 1999). When needed, antibiotics were used at the following concentrations: 100 μg/mL spectinomycin (Spec), 100 μg/mL chloramphenicol (Cm). A portion of the bacterial colonies grown in LB medium was stored at −80°C with 15% glycerol.

**TABLE 1:** Bacterial strains and plasmids used in this study.

	**Description**	**Source or reference**
**Strains**		
*R. anatipestifer* RA-YM	*R. anatipestifer* wild-type strain (serotype 1)	Lab collection
*R. anatipestifer* ATCC 11845	Standard strain of *R. anatipestifer*	Purchased at Guangzhou Microbial Preservation Center
*E. coli* X7213	Thi-1 thr-1 leuB6 glnV44 fhuA21 lacY1 recA1 RP4-2-Tc:Mu λpir ΔasdA4 Δzhf-2:Tn10	Lab collection
**Plasmids**		
pIC333	Source of Spec^R^ cassette	Lab collection
pRE112	Suicide vector, sacB mobRP4 R6K ori Cm^R^	Life Technology
pRE112- RAint	Suicide vector with 10K GI integrase and attachment site	This study
pRE112-Spec-RAint	pRE112- RAint vector with Spec^R^	This study
pRE112-Spec-eGFP-RAint	pRE112-Spec-RAint with Spec promoter and eGFP CDS	This study

### PCR Amplification of GI Integrase and Integration and Excision Reaction

Primers used in this study are listed in Table 2. The existence of GIs in *R. anatipestifer* ATCC11845, *R. anatipestifer* RA-YM, and 20 clinical isolates was determined by the PCR (polymerase chain reaction) amplification of the integrase, a marker gene, using int1 F/R primer pair. Genomic island integration and excision were also determined using PCR amplification, with two pairs of primers, RAGI1 1F/1R, RAGI1 2F/2R primer pairs for integration, and RAGI1 1F/2R, RAGI1 2F/1R primer pairs for excision. The amplification reactions were performed in a T100^TM^ Thermal Cycler (Bio-Rad, California, United States). The reaction mixture included 10 μL 2 × MonAmp^TM^ Taq Mix (Code No. RN03001M), 2 μL (100 ng/μL) *R. anatipestifer* DNA template, 1 μL of each primer (10 pmol/μL), and the volume was made up to 20 μL with water. The PCR program was as follows: denaturation at 95°C for 5 min followed by 30 cycles of denaturation at 94°C for 15 s, annealing at 60°C for 15 s, and extension at 72°C for 1 min, and a final extension at 72°C for 5 min. The PCR products were separated and detected by 0.8% agarose gel electrophoresis.

**TABLE 2:** Primers used in this study.

**Name**	**Sequence (5’-3’)**
int1 F	ATGGCAAATATCATTTTTTACCTAAGAGGCAC
int1 R	TTAGTTTAGTTGTTCAGGGGTAATGTTC
RAGI1 1F	GTATTTCCGTAGTCGCTTTGAGGATTCCTTCATCTTG
RAGI1 1R	TTTCATTTATTCAAAATGAGGGTTAAAATGAGTAAAAC
RAGI1 2F	CCACTTATCACAACGGTACTCTTAGCCAAATTAAAG
RAGI1 2R	CAATTTTCAGAATGGATAACAAAGGGGGTGTTTATA
RAint1 F	TCACGCGT TTTCATTTATTCAAAATGAGGGTTAAAATGAG
RAint1 R	GTGCATGC CTTAAAGTATTAGTTTAGTTGTTCAGGGGTAATGTTC
Spec^R^ F	TGCGGTACC TATCAATCTACTGGACAGTAGTTTTAAAAG
Spec^R^ R	TCACGCGT AAAACAACTTCAAAACAGTGGAACGAAAAC
Pspec R	CAGCTCCTCGCCCTTGCTCACCAT GATTTCACCTCGTTGATTATGTTCATATAA
eGFP F	GAACATAATCAACGAGGTGAAATC ATGGTGAGCAAGGGCGAGGAGCTGTTCACC
eGFP R	TCACGCGT TTACTTGTACAGCTCGTCCATGCCGAG

*The underline sequences indicated restriction sites.*

### Construction of Integration Plasmids With Integrase and Attachment Site

To study the integration and excision of GIs, the integration plasmid, containing the integrase and attachment site, was constructed. The mini integration island was generated with precise site-specific integration via the inclusion of the attachment site attP (116 bp) in the left and the right integrase coding sequence (1356 bp). The integration element was amplified with the primer pair RAint1 F and RAint1 R. The clone fragment with *Mlu*I and *Sph*I restriction enzyme sites was ligated into the suicide plasmid pRE112 using T4 DNA ligase (Invitrogen^TM^, Code No. 15224017). The resulting recombinant plasmid was called as pRE112-RAint. The chloramphenicol resistance cassette of plasmid pRE112 was not functional in *R. anatipestifer*, so the spectinomycin resistance cassette was cloned into pRE112-RAint with primers Spec^R^ F/R (*Kpn*I and *Mlu*I). The new plasmid was called as pRE112-Spec-RAint.

### Suicide Recombinant Vector Integration Into *R. anatipestifer*

The integration of the suicide recombinant vector into *R. anatipestifer* was carried according to our previously described method (Wang et al., 2017). *R. anatipestifer* RA-YM was selected as the receptor strain, as it did not have the 10K GI but had the 53 bp attachment site attB. The pRE112-Spec-RAint plasmid was transformed into the donor strain *E. coli* X7213. The *R. anatipestifer* YMint strain was confirmed by PCR, and verification of integration and excision function of the recombinant strains.

### Integrase-Mediated Expression of eGFP in *R. anatipestifer*

To determine whether the integration plasmid could express foreign proteins, the Spec resistance protein promoter was cloned by Spec^R^ F/Pspec R primer pairs, fused with the eGFP coding sequence, was linked to pRE112-Spec-RAint with *Kpn*I and *Mlu*I by overlapping extension PCR. The expression vector was named pRE112-Spec-eGFP-RAint. The integration of the expression vector into *R. anatipestifer* was performed according to the mentioned above. The protein was detected by western blotting. The detail methods are as follows (Guo et al., 2016): Sample preparation, eGFP recombinant *R. anatipestifer* RA-YM and wild type *R. anatipestifer* RA-YM cultured in TSB overnight. 1 mL bacterial suspension was centrifuged for 3 min at 5000 rpm and washed twice with 1 mL PBS at pH 7.4. Bacteria precipitation were resuspended by 40 μL PBS, and mixing 40 μL 2 × SDS sampling buffer (Beyotine, P0015B). The samples boiled for 10 min. SDS-PAGE, 12.5% SDS-page gel maked by PAGE Gel Fast Preparation Kit (EpiZyse, PG 113). Load 20 μL samples and 10 μL protein marker (Thermo Fisher Scientific, 26616) onto the gel. 80 V to run for 30 min, and increase to 120 V for 1 h 30 min. Electrotransfer, Wet transfer systems for 60 min at 100 mA. Immunoblotting, pH 7.6 TBST (150.57 mM NaCl, 24.76 mM Tris base, 0.1% Tween-20) containing 5% skim milk (Difco) for Blocking membrane at 4°C overnight. eGFP rabbit polyclonal antibody (Proteintech, 50430-2-AP) 1:2000 in TBST and membrane was incubated at room temperature for 3 h, and washed 3 times with TBST for 5 min. The membrane was incubated at room temperature for 2 h with HRP goat anti-rabbit IgG (ABclonal, AS014) diluted at 1:5000 in TBST and then washed 3 times with TBST for 10 min. ECL (ABclonal, RM00021) for signal detection in chemiluminescence imaging system (United States, BIO-RAD).

To observe fluorescence, the eGFP recombinant *R. anatipestifer* RA-YM and wild type *R. anatipestifer* RA-YM cultured in TSB overnight. 1 mL bacterial suspension was centrifuged for 3 min at 5000 rpm and washed twice with 1 mL PBS at pH 7.4. Bacteria precipitation were resuspended by 1 mL PBS. 10 μL bacterial fluid uniformly to the glass slide and dry at room temperature (Xie et al., 2017). The fluorescence in the bacteria was observed and photographed by the confocal laser-scanning microscope (Germany, Leica).

## Results

### Analysis of the 10K GI in *R. anatipestifer*

**FIGURE 1:**
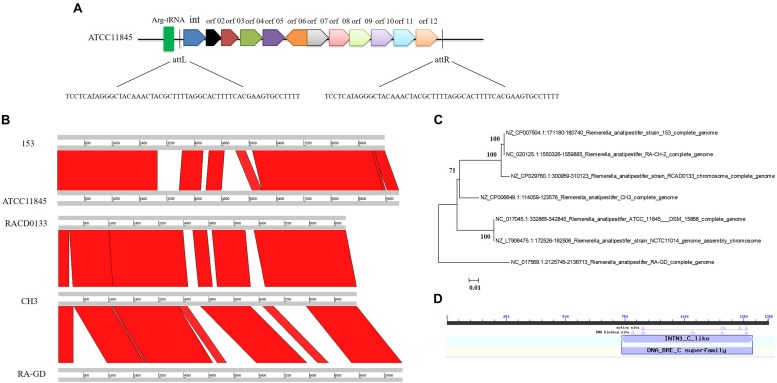
10K genomic island in *Riemerella anatipestifer.* **(A)** Analysis of the 10K genomic island related genes in *R. anatipestifer.* The 7 *R. anatipestifer* GIs sequence were compared **(B)** and phylogenetic tree was constructed containing 7 10K GI sequences **(C)**. **(D)** Bioinformatics analysis the conserved domain of integrase in 10K genomic island.

*Riemerella anatipestifer* ATCC11845 strain was analyzed by the IslandViewer4 server, and a 10K GI was identified. The 10K GI included 12 open reading frames (showed in Supplementary Table S1) and a 53 bp (TCCTCATAGGGCTACAAACTACGCTTTTAGGCACTTTTC ACGAAGTGCCTTTT) directed repeat (attL and attR), located at two flanks of GI, the GI next to arginine (Arg)-tRNA and downstream of the Arg-tRNA (Figure 1A). Genome-wide comparative analysis of the 33 genomic sequences of the *R. anatipestifer* strains revealed that 20 *R. anatipestifer* strains had 10K GIs, and they are RCAD0133, RCAD0111, DSM15868, ATCC 11845, NCTC 11014, RCAD0142, RCAD0131, CH3, 17CS0503, RA-CH-2, 153, RCAD0188, RCAD0183, RCAD0134, RCAD0124, RCAD0122, RA-GD, RA-SG, RA-JLLY, and RA2. Seven 10K GI sequences were compared and the phylogenetic tree was constructed (Figures 1B,C). Twenty 10K GI sequences were compared and the phylogenetic tree was constructed in Supplementary Figure S1. *R. anatipestifer* ATCC11845 and *R. anatipestifer* NCTC11014 are the same strain from different collection centers, and have the same GI sequence. The other 5 GIs had varying degrees of deletions and insertions compared to *R. anatipestifer* ATCC11845. Among the 12 ORFs, an integrase was predicted. The detail information of all the twenty 10K GIs found in *R. anatipestifer* showed in Supplementary Table S2. The Phyre2 web was used for the prediction of the protein structures (Kelley et al., 2015), and homologous amino acid sequences were identified by searching the GenBank database using BLASTX. The integrase belonged to the tyrosine integrase family, with an intN1_C_like domain (Figure 1D). Our results demonstrated that the 10K GIs differ in the *R. anatipestifer* strains. The integrase was found to be highly conserved in *R. anatipestifer.* The Amino acid sequence alignment results were showed in Supplementary Figure S2.

### Integration and Excision of the *R. anatipestifer* 10K GI

**FIGURE 2:**
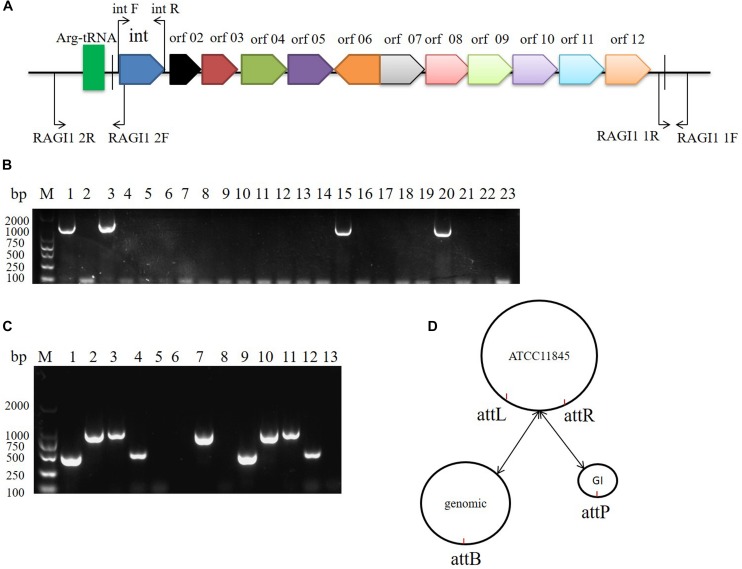
10K genomic island integration and excision in *R. anatipestifer*. **(A)** Primer designed for detecting of 10K GI, integration and excision. **(B)** Detecting of 10K GI in *R. anatipestifer* clinical isolated strain with primer pair int1 F/R. Lane 1, *R. anatipestifer* ATCC11845 strain; Lane 2, *R. anatipestifer* RA-YM strain; Lane 3–22, 20 clinical isolated strains No. 1–20; Lane 23, negative control. **(C)** Detecting of genomic island integration and excision, M: DL2000 DNA marker. Lane 1, DNA fragment was amplified with RAGI1 1F/1R primer pair from *R. anatipestifer* ATCC11845; Lane 2, DNA fragment was amplified with RAGI1 2F/2R primer pair from *R. anatipestifer* ATCC11845; Lane 3, DNA fragment was amplified with RAGI1 1F/2R primer pair from *R. anatipestifer* ATCC11845; Lane 4, DNA fragment was amplified with RAGI1 2F/1R primer pair from *R. anatipestifer* ATCC11845; Lane 5–8, DNA fragment was amplified with same primer from *R. anatipestifer* RA-YM; Lane 9–12, DNA fragment was amplified with same primer from *R. anatipestifer* clinical isolated strain No.1. Lane 13, negative control. **(D)** 10K GI integration and excision model. The dynamic balance of 10K genomic island integration and excision in *R. anatipestifer* ATCC11845 genome.

The 10K GI in *R. anatipestifer* ATCC11845, *R. anatipestifer* RA-YM, and the 20 clinical *R. anatipestifer* isolates was detected by PCR with the 1356 bp integrase gene marker (Figure 2A). The results showed that *R. anatipestifer* ATCC11845 and 3 clinical isolates had the 10K GI, whereas *R. anatipestifer* RA-YM and 17 clinical isolates lacked the 10K GI (Figure 2B). The primer pair RAGI1 1F/1R was used for detecting the 10K GI integration location. The 905 bp PCR products were sequenced (Supplementary Table S3), and the results showed a 10K GI with a 53 bp attL sequence, which is one of the integration sites. This was consistent with the genome prediction. The results for the excision of the 10K GI from the genome demonstrated that the 10K GI could automatically excise from the chromosome and form a circular extrachromosomal DNA (Figure 2C), thus establishing a dynamic equilibrium of integration, and excision. Figure 2D is a schematic model of the integration and excision processes.

### Integrase-Mediated Integration and Excision

**FIGURE 3:**
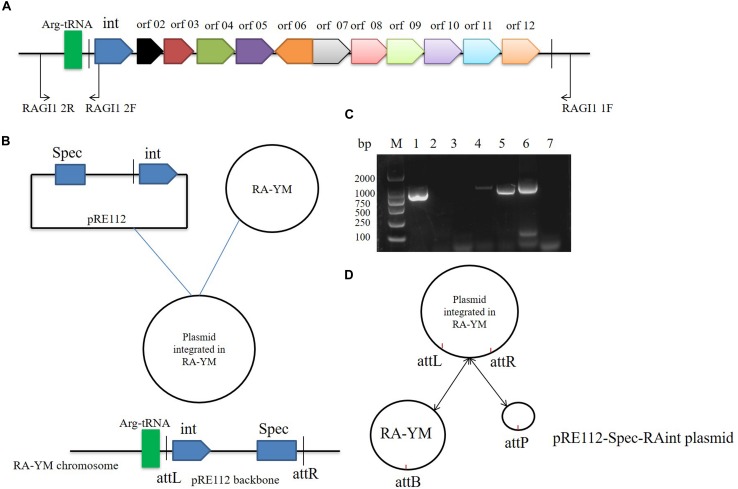
Integrase mediated integration and excision. **(A)** Primer designed for integrase cloned. **(B)** The vector construction and integration mechanism. **(C)** Integrase mediated integration and excision in *R. anatipestifer* RA-YM strain and *R. anatipestifer* YMint strain. Lane 1, PCR for integration with RAGI1 1F/2R primer pair in *R. anatipestifer* RA-YM strain; Lane 2, PCR for excision with RAGI1 2F/2R primer pair in *R. anatipestifer* RA-YM strain; Lane 3, PCR for Spec^R^ resistant gene with Spec^R^ F/R primer pair in *R. anatipestifer* RA-YM strain; Lane 4, PCR for integration with RAGI1 1F/2R primer pair in *R. anatipestifer* YMint strain; Lane 5, PCR for excision with RAGI1 2F/2R primer pair in *R. anatipestifer* YMint strain; Lane 6, PCR for Spec^R^ resistant gene with Spec^R^ F/R primer pair in *R. anatipestifer* YMint strain; Lane 7, negative control; M, DL2000 DNA marker. **(D)** Integrase mediated integration and excision model. The dynamic balance of pRE112-Spec-RAint suicide plasmid integration and excision in *R. anatipestifer* RA-YM genome.

We studied the molecular mechanisms of integration and excision in *R. anatipestifer* RA-YM. A 1716 bp DNA fragment was cloned into the suicide plasmid successfully. The insert sequence included single 53 bp direct repeat and the integrase ORF along with the promoter. The recombinant suicide vector was transferred from *E. coli* X7213 strain to the *R. anatipestifer* RA-YM strain via conjugal transfer. The recombinant strain was selected using Spec antibiotic plate. The vector construction and integration mechanism are shown in Figures 3A,B, respectively. Integration and excision were detected by PCR in the recombinant strains. The PCR products were sequenced and identified using BLASTX; the results indicated that plasmid was integrated into *R. anatipestifer* RA-YM, and the insert site was consistent with the *R. anatipestifer* ATCC11845 strain. We also found that the integrase not only mediated integration but also excision from the recombinant strain (Figures 3C,D). Our results suggested that the 10K GI integrase has both integration and excision functions.

### Integrase-Mediated Heterologous Gene Expression in *R. anatipestifer*

**FIGURE 4:**
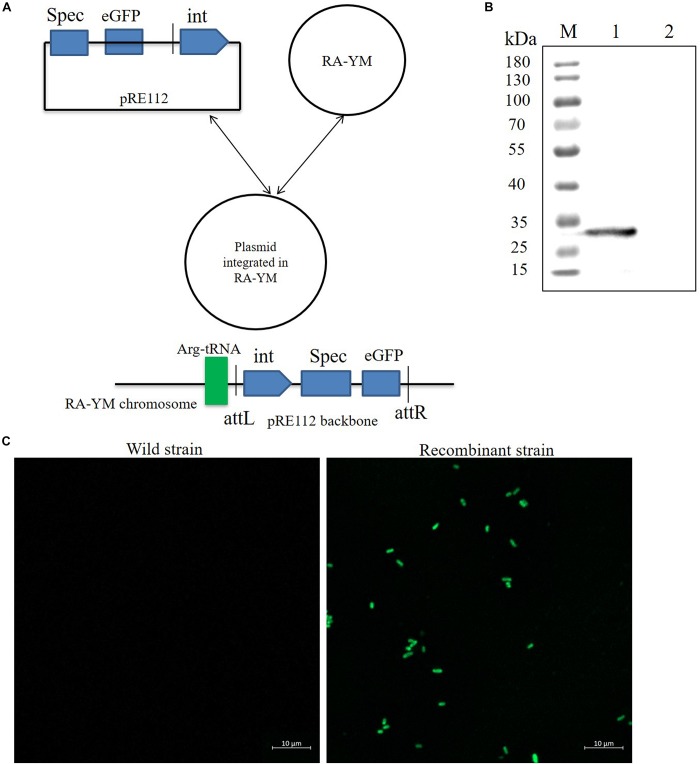
Integrase mediated heterologous gene expression in *R. anatipestifer* RA-YM strain. **(A)** The eGFP integration vector construction diagram. **(B)** Western blot for detecting expression of eGFP protein. The eGFP fluorescent protein were observed by confocal microscope in *R. anatipestifer* RA-YM wild strain and recombinant strain of integrating eGFP expression **(C)**.

To develop a gene expression system for *R. anatipestifer*, we used the integration plasmid as described above. A Spec promoter with eGFP coding sequence was spliced by overlapping extension PCR. The eGFP protein expression vector was named as pRE112-Spec-eGFP-RAint. The vector construction diagram is shown in Figure 4A. The recombinant expression vector was transferred from *E. coli* X7213 strain to *R. anatipestifer* RA-YM strain via conjugal transfer. The eGFP protein was observed by western blotting and confocal microscope (Figures 4B,C). Our results indicated that integrase could mediate eGFP expression in *R. anatipestifer*. This system could be useful in the ectopic expression of the deleted genes, as a complementary plasmid, in *R. anatipestifer*.

## Discussion

Up to now, 33 strains of *R. anatipestifer* have been sequenced, but no detailed GIs have been reported. Through bioinformatics analysis, we found a 10K insertion sequence in *R. anatipestifer* ATCC11845. The GIs share several features: (1) large DNA segments, usually between 10 and 200 kb (Song et al., 2012), (2) the GC content usually differ from the rest of the chromosome, (3) GIs are often inserted at tRNA genes (Zhang et al., 2011), (4) GIs are often flanked by perfect direct repeats (DR), (5) GIs often harbor integrases, and (6) GIs often carry transposons, insertion sequences, and other mobile elements as well as functional gene clusters. A 53 bp perfect DR sequence was located at the flank of the insertion sequence. There was a tyrosine integrase next to the Arg-tRNA. Prophage also had the same characteristics, and no plaque was induced by mitomycin and UV (data not shown). This insertion sequence was called the 10K GI.

Genome-wide comparative analysis revealed that out of the 33 *R. anatipestifer* strains, 20 strains had the 10K GI. Genomes of bacterial species can evolve through a variety of processes, including mutations, rearrangements, or horizontal gene transfer (Juhas et al., 2009). Horizontal gene transfer plays an important role in microbial evolution, enabling the acquisition of new genes and phenotypes. The comparison between the functional and phylogenetic evolution of the horizontal gene transfer events showed that it influences the genome of *R. anatipestifer* (Liu et al., 2019). Integration and conjugation elements (ICEs) are modular mobile genetic elements integrated into the host genome, which reproduce passively during chromosome replication and cell division (Murphy and Boyd, 2008). The ICEs belong to a group of GIs and are usually found to be integrated into the host bacterial chromosomes and can be removed to form a round product as a conjugate substrate (Menard and Grossman, 2013). We detected the integration and excision by PCR, and the results showed that the 10K GI could integrate and excise automatically. Twelve ORFs were coded by the 10K GI. Most proteins were predicted as hypothetical proteins. The transfer of ICEs involves the interaction of various proteins; whether the GI undergoes a horizontal metastasis needs further verification.

Integrases play a core role in GIs and are similar to a bacteriophage. The mechanism of site-specific recombination is similar to bacteriophage integration. Integrases interact with attL and attR direct repeats of the GIs, mediating excision from the host chromosomes, and formation of the cyclization intermediates. The Cre-loxP site-specific recombination system was encoded by the *E. coli* λ phage P1. The process of integration and excision only requires the Cre integrase. The Lambda site-specific recombination system was encoded by the *E. coli* λ phage. The process of integration and excision requires lambda integrase as well as Xis and integration host factors. A 1716 bp DNA fragment, including the integrase and attP site, was integrated into *R. anatipestifer* RA-YM, and the integration and excision were consistent with the *R. anatipestifer* ATCC11845 strain.

The 10K GI has an intN1_C_like domain. The intN1 was identified as an integrase of the non-replicative *Bacteroides* unit 1 (NBU1) (Rajeev et al., 2006, 2007). The excision of NBU1 is a complex process and needs other proteins that are involved in transposon interaction with intN1 (Wood et al., 2013). In contrast, the excision of the 10K GI did not need GI encoded proteins. The results indicated that the integrase from the 10K GI not only helped in integration but also in excision. The integrase was similar to Cre and Flp; integration and excision only needed tyrosine integrase. We will further study the biological functions of this integrase at the molecular level.

The shuttle plasmid replication element, replicon, originated in the *R. anatipestifer* endogenous plasmid (Guo et al., 2017; Feng et al., 2018). In this study, we developed a site-directed integration plasmid for heterologous gene expression in *R. anatipestifer*, and the results showed that protein could express in *R. anatipestifer* strains, lacking the 10K GI; for example *R. anatipestifer* CH-1, *R. anatipestifer* RA-YM, *R. anatipestifer* Yb2, and *R. anatipestifer* RA1.

In summary, our research showed that the 10K GI is widely found in the *R. anatipestifer* genome. We had first reported the GI integration and excision function in *R. anatipestifer*. We successfully constructed an integration plasmid by using the integrase and attP elements and identification the integrase responsible for the precise integration and excision in *R. anatipestifer.* We elucidated the molecular mechanism of the 10K GI integration and excision. Furthermore, we provided a new method for the heterologous gene expression and the construction of complementary strain. In conclusion, this was a comprehensive study on the integration and excision of the *R. anatipestifer* 10K GI, which laid the foundation for horizontal gene transfer and the evolution of *R. anatipestifer* genome.

## Data Availability

All datasets generated for this study are included in the manuscript and/or the Supplementary Files.

## Author Contributions

ZL: funding acquisition, supervision, validation, and writing (review and editing). YW: investigation, visualization, and writing – original draft. YW, YZ, YC, and ZS: methodology. SH, SL, ML, XM, YX, DS, and DB: project administration. ZZ: resources.

## Conflict of Interest Statement

The authors declare that the research was conducted in the absence of any commercial or financial relationships that could be construed as a potential conflict of interest.

## Footnotes

1 www.ncbi.nlm.nih.gov/genome/genomes/2536?

## Supplementary Material

FIGURE S1 Twenty 10K GI sequences were compared and the phylogenetic tree was constructed.

FIGURE S2 The conserved integrase amino acid sequence alignment results.

TABLE S1 *Riemerella anatipestifer* ATCC 11845 10K GI ORFs list.

TABLE S2 10K GI in twenty *Riemerella anatipestifer* strains.
