# Supplementary figures and images for: Identification of an Integrase That Responsible for Precise Integration and Excision of Riemerella anatipestifer Genomic Island
Source: Front Microbiol. 2019 Sep 20;10:2099. doi: 10.3389/fmicb.2019.02099 (PMC6764341; doi:10.3389/fmicb.2019.02099)

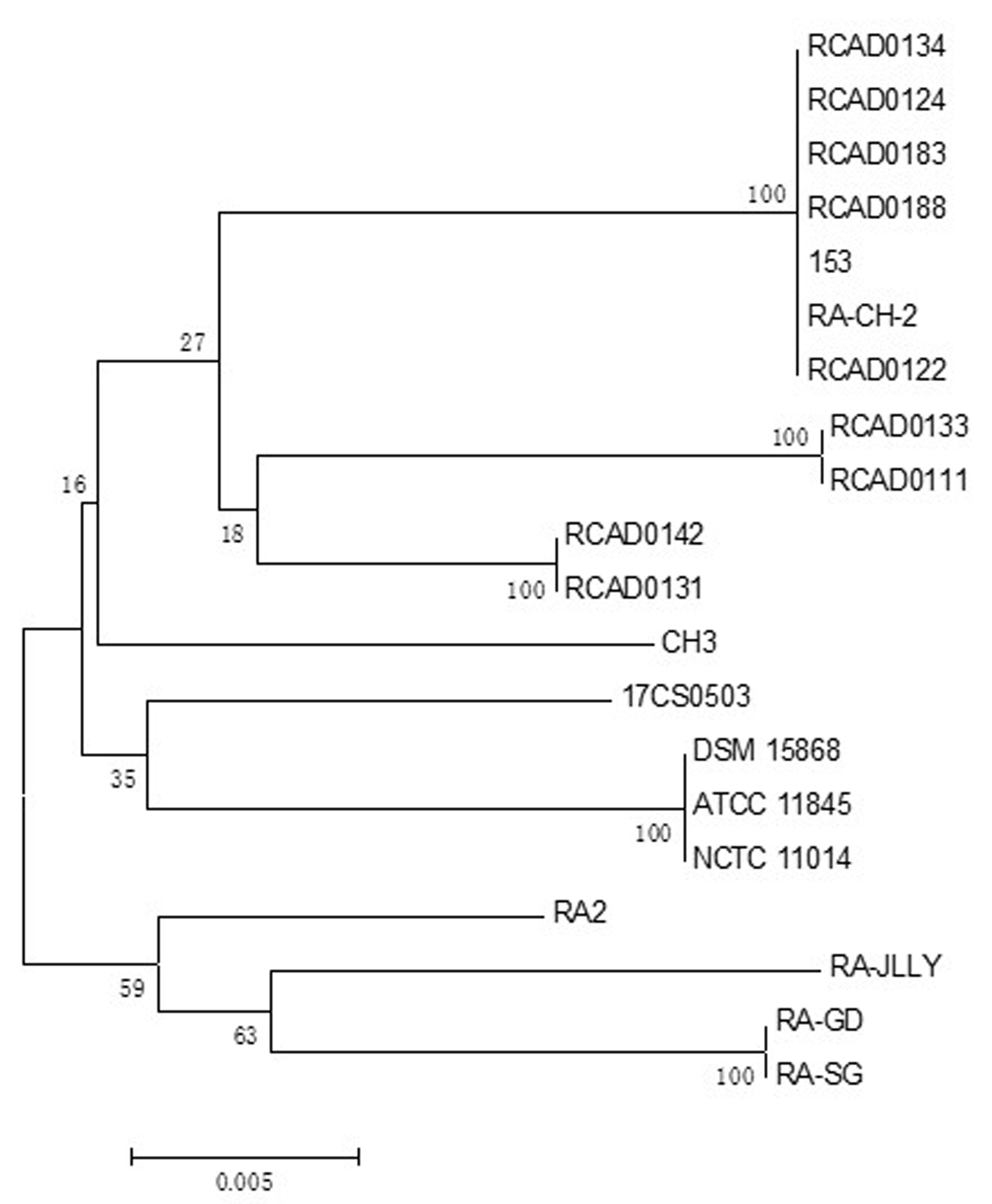

Supplement: FIGURE S1 — Twenty 10K GI sequences were compared and the phylogenetic tree was constructed. [file Image_1.TIF]

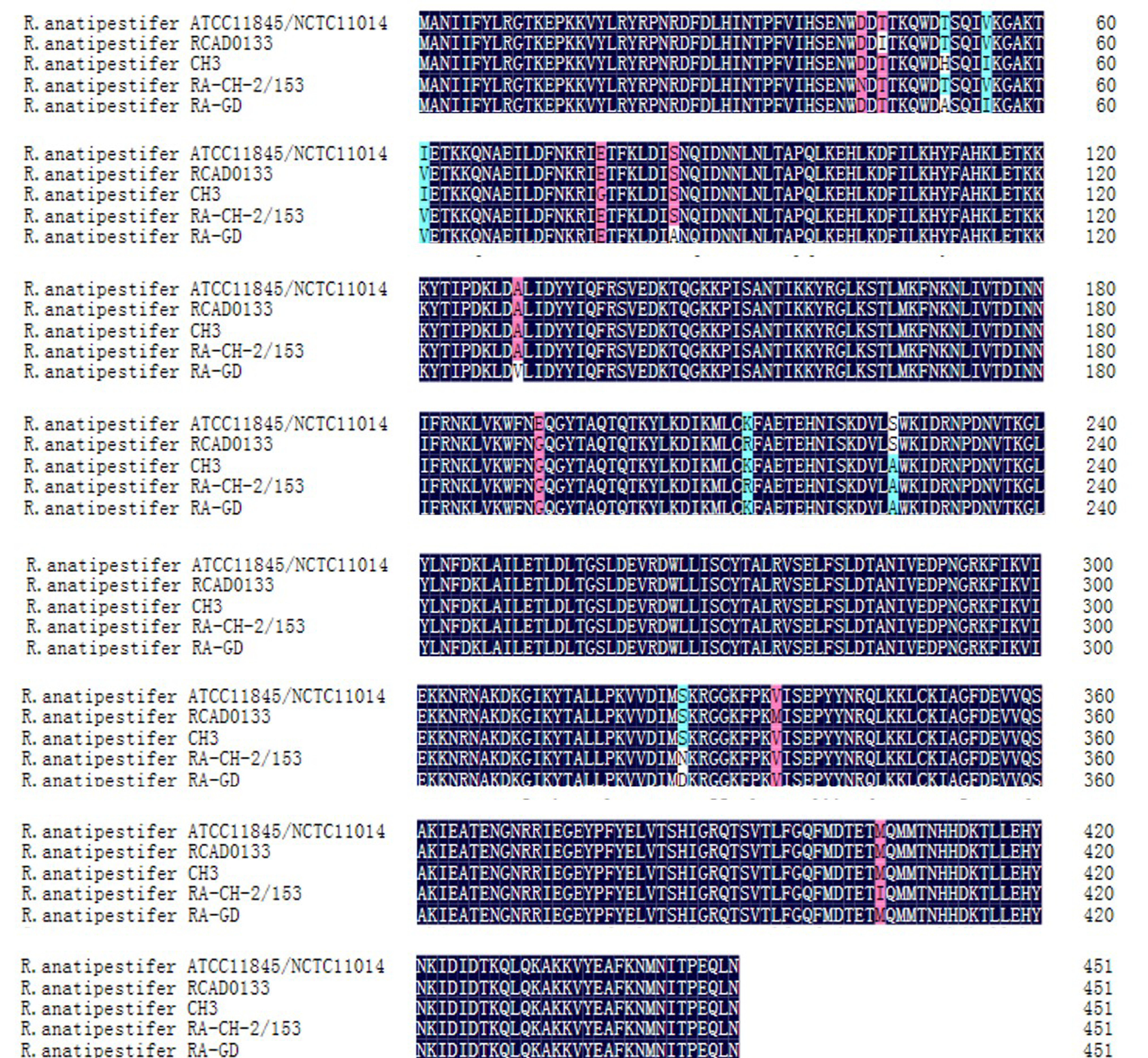

Supplement: FIGURE S2 — The conserved integrase amino acid sequence alignment results. [file Image_2.TIF]
