# Supplementary material for: Identification of an Integrase That Responsible for Precise Integration and Excision of Riemerella anatipestifer Genomic Island
Source: Front Microbiol. 2019 Sep 20;10:2099. doi: 10.3389/fmicb.2019.02099 (PMC6764341; doi:10.3389/fmicb.2019.02099)
Supplement: TABLE S1 — Riemerella anatipestifer ATCC 11845 10K GI ORFs list. [file Table_1.docx]

Supplementary Table S1

*Riemerella anatipestifer* ATCC 11845 10K GI ORFs list

| protein_ID | Locus | Length (bp) | Product |
| --- | --- | --- | --- |
| [AFD55360.1](https://www.ncbi.nlm.nih.gov/protein/380459676) | RA0C_0375 | 1356 | integrase family protein |
| [AFD55361.1](https://www.ncbi.nlm.nih.gov/protein/380459676) | RA0C_0376 | 1062 | filamentation induced by camp protein fic |
| [AFD55362.1](https://www.ncbi.nlm.nih.gov/protein/380459676) | RA0C_0377 | 744 | hypothetical protein |
| [AFD55363.1](https://www.ncbi.nlm.nih.gov/protein/380459676) | RA0C_0378 | 288 | DNA binding domain protein |
| [AFD55364.1](https://www.ncbi.nlm.nih.gov/protein/380459676) | RA0C_0379 | 612 | anta/antb antirepressor domain protein |
| [AFD55365.1](https://www.ncbi.nlm.nih.gov/protein/380459676) | RA0C_0380 | 315 | anta/antb antirepressor domain protein |
| [AFD55366.1](https://www.ncbi.nlm.nih.gov/protein/380459676) | RA0C_0381 | 219 | hypothetical protein |
| [AFD55367.1](https://www.ncbi.nlm.nih.gov/protein/380459676) | RA0C_0382 | 309 | hypothetical protein |
| [AFD55368.1](https://www.ncbi.nlm.nih.gov/protein/380459676) | RA0C_0383 | 438 | hypothetical protein |
| [AFD55369.1](https://www.ncbi.nlm.nih.gov/protein/380459676) | RA0C_0384 | 531 | hypothetical protein |
| [AFD55370.1](https://www.ncbi.nlm.nih.gov/protein/380459676) | RA0C_0385 | 423 | hypothetical protein |
| [AFD55371.1](https://www.ncbi.nlm.nih.gov/protein/380459676) | RA0C_0386 | 1383 | hypothetical protein |
