# Supplementary material for: Identification of an Integrase That Responsible for Precise Integration and Excision of Riemerella anatipestifer Genomic Island
Source: Front Microbiol. 2019 Sep 20;10:2099. doi: 10.3389/fmicb.2019.02099 (PMC6764341; doi:10.3389/fmicb.2019.02099)
Supplement: TABLE S2 — 10K GI in twenty Riemerella anatipestifer strains. [file Table_2.DOCX]

Supplementary Table S2

10K GI in twenty *Riemerella anatipestifer* strains

| Strain name | Accession number | Position | Length(bp) | Total CDS |
| --- | --- | --- | --- | --- |
| RCAD0133 | NZ_CP029760 | 300959-310123 | 9165 | 9 |
| RCAD0111 | NZ_LUDR01000015 | 31785-41913 | 10129 | 12 |
| DSM 15868 | NC_014738 | 172541-182521 | 9981 | 12 |
| ATCC 11845 | NC_017045 | 332865-342845 | 9981 | 12 |
| NCTC11014 | LT906475 | 172526-182506 | 9981 | 12 |
| RCAD0142 | NZ_LUDG01000001 | 139009-149192 | 10184 | 13 |
| RCAD0131 | NZ_LUDS01000001 | 139081-149264 | 10184 | 13 |
| CH3 | NZ_CP006649 | 114059-123576 | 9518 | 10 |
| 17CS0503 | NZ_PKKR01000008 | 10279-21116 | 10838 | 14 |
| RA-CH-2 | NC_020125 | 606437-615996 | 9560 | 11 |
| 153 | NZ_CP007504 | 171180-180740 | 9561 | 11 |
| RCAD0188 | NZ_LUDH01000001 | 642111-651670 | 9560 | 11 |
| RCAD0183 | NZ_LUDK01000001 | 139009-148568 | 9560 | 11 |
| RCAD0134 | NZ_LUDO01000009 | 29868-39427 | 9560 | 11 |
| RCAD0124 | NZ_LUDQ01000009 | 29868-39392 | 9525 | 11 |
| RCAD0122 | NZ_LUDU01000009 | 29846-39405 | 9560 | 11 |
| RA-GD | NC_017569 | 2125745-2136713 | 10969 | 14 |
| RA-SG | NZ_KB206037 | 704851-715807 | 10957 | 14 |
| RA-JLLY | NZ_LAVB01000007 | 117914-128182 | 10269 | 12 |
| RA2 | NZ_QEWX01000017 | 2585-14165 | 11581 | 16 |
